# Prevalence and determinants of anaemia among pregnant women in a high malaria transmission setting: a cross-sectional study in rural Burkina Faso

**DOI:** 10.11604/pamj.2024.47.2.40612

**Published:** 2024-01-03

**Authors:** Moussa Lingani, Serge Henri Zango, Innocent Valéa, Sékou Samadoulougou, Moussa Abdel Sanou, Hermann Sorgho, Edmond Sawadogo, Michèle Dramaix, Philippe Donnen, Robert Annie, Halidou Tinto

**Affiliations:** 1 Institut de Recherche en Sciences de la Santé Direction Régionale du Centre Ouest (IRSS/DRCO), Nanoro, Burkina Faso,; 2 Evaluation Platform on Obesity Prevention, Quebec Heart and Lung Institute Research Center, Quebec City, Canada,; 3 École de Santé Publique, Université Libre de Bruxelles, Route de Lennik, Bruxelles, Belgique,; 4 Epidemiology and Biostatistics Research Division, *Institut de Recherche Expérimentale et Clinique, Université Catholique de* Louvain, Brussels, Clos Chapelle-aux-Champs, Brussels, Belgique

**Keywords:** Anaemia, pregnancy, malaria, rural area, Burkina Faso

## Abstract

**Introduction:**

anemia, the commonest nutritional deficiency disorder among pregnant women in sub-Saharan Africa, is associated with severe peripartum complications. Its regular monitoring is necessary to timely inform clinical and preventive decision-making. The aim of this study was to assess the prevalence and determinants of anemia among pregnant women in rural areas of Burkina Faso.

**Methods:**

between August 2019 and March 2020, a cross-sectional study was conducted to collect maternal sociodemographic, gynaeco-obstetric, and medical characteristics by face-to-face interview or by review of antenatal care books. In addition, maternal malaria was diagnosed by standard microscopy and the hemoglobin levels (Hb) measured by spectrophotometry. The proportion of anaemia (Hb<11.0 g/dL), moderate (7.0<Hb<9.9 g/dL) and severe (Hb<7.0 g/dL) anaemia were determined. The maternal factors associated with anaemia were identified using regression models with likelihood ratio tests. A p-value < 0.05 was considered statistically significant.

**Results:**

of 594 pregnant women assessed, the mean hemoglobin level (± standard deviation) was 10.7 (±0.1) g/dL, and the prevalence of anemia was 54.4% (323/594). The proportion of moderate, and severe anemia among pregnant women was 49.2% (95% CI: 45.1%-53.2%), and 5.2% (95% CI: 3.7%-7.3%) respectively. Multivariate analysis showed that the young maternal age (<20 years old) (adjusted OR (aOR): 1.5, 95% CI: 1.1-2.3) and the presence of malaria (aOR: 2.0, 95% CI: 1.3-3.2) were independently associated with the presence of maternal anemia.

**Conclusion:**

anemia remains common in the study setting and interventions to strengthen malaria prevention in pregnancy, particularly among young adolescent pregnant women, are required to prevent maternal anemia.

## Introduction

Anemia in pregnancy, defined as the hemoglobin concentration of less than 11g/dL [1], is associated with severe adverse reproductive outcomes such as preterm delivery (PD), low birth weight (LBW), and decreased iron stores for the neonates [1]. Anemia impairs the capacity of blood to transport oxygen around the body and is a key indicator of poor nutrition and health [2], an important contributor to maternal morbidity and mortality as it leads to more than 115 000 maternal deaths each year in developing countries [3]. It is also associated with an increased risk of miscarriage [4], prematurity, stillbirth, low birth weight and consequently perinatal mortality [5]. Globally, anemia affects half a billion reproductive age women [6], and in Burkina Faso the prevalence varies between 56.6% and 61.2% despite the adoption of numerous prevention strategies within the antenatal care package [7]. The causes of anemia have remained multifactorial in sub-Saharan Africa, and malaria is a leading cause, particularly in the rural areas [8-10]. In area of endemic malaria transmission, the World Health Organization (WHO) developed the intermittent preventive treatment of malaria in pregnancy using sulfadoxine-pyrimethamine (IPTp-SP) and a guideline for daily folic acid and iron supplementation for countries aiming to reduce malaria and iron deficiency related anaemia [6,11]. This guideline provides global, evidence-informed recommendations on daily iron and folic acid supplementation and IPTp-SP as public health interventions aiming to improve pregnancy outcomes and reduce anemia in pregnancy [6,11]. The strategies were shown effective in reducing the risk of anemia and low birth weight among pregnant women in malaria endemic settings [12]. These strategies have been widely used during the last two decades in Burkina Faso [13,14], however, anaemia and its consequences have remained common in the country, mainly attributable to logistic, budget, poor utilization of antenatal care services, inadequate counselling, poverty of populations, and low compliance problems [15]. Novel approaches or a readjustment of existing strategies are thus required for improved prevention strategies [16]. The scarcity of epidemiological data on anaemia during pregnancy has, however, been an obstacle for adequate decision-making particularly in the rural area of the country, thus detailed information is required. The aim of this study was to determine the prevalence and determinants of anemia among pregnant women visiting maternity services during their third trimester of pregnancy in the health district of Yako in northern Burkina Faso.

## Methods

**Study design and setting:** the present cross-sectional study was conducted in the Yako health district (YHD) catchment area in rural settings of Burkina Faso from August 2019 to March 2020 after the study protocol was approved by the National Ethics Committee (clearance certificate number 2018-7-096). Pregnant women who visited the selected health centres during their third trimester of pregnancy were assessed for inclusion. In the Yako health district, malaria transmission is holo-endemic with a marked seasonal transmission during the wet and rainy season (July-November) [17]. The health district area has a total population of 424 577 inhabitants, and an estimated 22 600 pregnancies are annually exposed to malaria [18].

**Sample size calculation:** the WHO global nutrition targets aim at achieving 50% reduction of anaemia in women of reproductive age by 2025 [2]. We assumed that anaemia prevention measures implemented since 2012 in the country have reduced by 30% the prevalence of anaemia (from 59% to 40%) by 2020 [7]. The Cochran formula was used to determine the appropriate sample size,


$$N=\frac{Z^{2}P(1-P)}{I^{2}}$$

where n is the sample size, z is the z-score that corresponds to the 95% confidence interval (1.96), p is the expected proportion, and i is the margin of error set at 5%. A sample size of 425 pregnant women was required, assuming 15% maximum of incomplete data.

**Study participants and sampling procedure:** pregnant women aged 15 to 45 years, that lived in all four health centers (district 4, the district 5, the district 6 and the Yako medical center) catchment area of Yako, within the Yako health district and who visited the health centre maternity wards during the third trimester of pregnancy, willing to adhere to the study procedures and who provided a written informed consent were recruited. They were not included if they could not be examined due to medical conditions that required reference to a larger hospital prior to their visit by study staff. Consecutive inclusion of pregnant women per selected health facility was carried out until the required sample size was completed.

**Variables and data sources/measurement:** variables related to socio-demographic, gynaeco-obstetric, medical characteristics, the use of bed net the previous night, the number of IPTp-SP doses, the level of education, as well as the pregnant women occupation were collected and recorded on a semi-structured questionnaire from the pregnant women health booklets or by person-to-person interview whenever the information was not available in the health documents. In addition, to gynaeco-obstetrical examination, maternal body weight, height, blood pressure, axillary temperature were measured by study clinicians. Malaria was detected by standard microscopy and the haemoglobin level was measured with a portable spectrophotometer (HemoCue, Ängelholm, Sweden). The outcome variable was anaemia defined as haemoglobin level from finger prick blood samples (Hb) <11g/dL. Explanatory variables included malaria (detection of *P. falciparum* asexual parasites by microscopy), non-optimal SP uptake (<3 doses), the number of prenatal visits, the gravidity (primigravid if first pregnancy, paucigravid if 2 - 4 pregnancies, and multigravida if ≥5), body mass index (BMI) derived by the maternal body weight in kilograms divided by the square of the height in meters (low if <18.5, normal or high if ≥18.5), history of miscarriage or stillbirth, high blood pressure (if systolic blood pressure ≥140 mmHg or diastolic blood pressure ≥90 mmHg), level of education (none, primary, secondary or plus), occupation (Unemployed, employed/self-employed), the use of bed net the previous night before the visit.

**Laboratory testing:** haemoglobin level was assessed from finger pricks´ blood samples with portable haemoglobinometer, HemoCue. For malaria screening, thick and thin blood smears, also taken from finger pricks, were stained with 5% Giemsa for 30 min and examined by two independent microscopists at 100x magnification using light microscopy. Parasite densities were calculated by counting the asexual parasites number per 200 whites blood cell (WBC), and assuming a WBC count of 8000 cells per µl of blood. When asexual parasites count was less than 100 per 200 WBC, counting was performed against at least 500 WBC. A smear was declared negative if no parasite was detected after the review of 1000 WBC or 100 fields containing at least 10 WBC per field. In the case of discrepancy (different species or count difference ≥50%), a third reading was required and the average of the two closest results was used as final count.

**Data management and statistical analysis:** data were double entered from the questionnaire onto a Redcap (Research Electronic Data Capture) electronic database and exported on Stata version 15 (Stata Corp. 2017, TX, USA) for cleaning and analysis. Descriptive statistics were performed to illustrate the basic characteristics of the study population. Frequencies were calculated for categorical variables, mean or median with respective standard deviations or interquartile ranges for numerical variables. Prevalence of anaemia was estimated as a proportion of pregnant women with haemoglobin level < 11 g/dL. Odds ratios (OR) with 95% confidence intervals (95% CI) were calculated by univariate logistic regression using likelihood ratio tests to assess the association between anaemia and maternal factors. Adjusted OR (aOR) were derived by backward multivariate logistic regression of factors which p-values were <0.1 at univariate analysis and retaining in the final model variables with p-values <0.1. The significance level was set at 5%. Participants with missing data for the main outcome were not included.

**Ethical approval and consent to participate:** the study was conducted in accordance with good clinical practice and all procedures involving the participants were approved by the national ethics committee of health science research of Burkina Faso (clearance certificate number 2018-7-096). In addition, all participants provided written inform consents prior to their recruitment. Cases of anaemia and malaria received free care according to the national treatment guidelines.

## Results

Of 684 participants assessed, this study included 594 (86.7%) of them, and 90 (13.2%) were not eligible. Reason for non-eligibility included missing haemoglobin data due to shortage of testing reagents on the day of visit (36), not third trimester of pregnancy (31), consent refusal (18), and not residing in the study area (5). Women were mainly unemployed (69.2%) with none or primary level of education (67.8%). Mean age was 24±6 (range; 15-42) years, and 22.9% were aged less than 20 years. The median number of pregnancies was two (interquartile range, IQR; 1-4). Regarding malaria prevention measures, 93% stated using Insecticide-treated nets (ITN) the night prior to their visit and the IPTp-SP uptake was 77.1%, 16.9%, 5.1% and 1% for 3 or more doses, 2 doses, one dose and no dose respectively. Malaria infection was detected in 17.5% (104/594) of participants (Table 1). The overall anaemia prevalence among women was 54.3% (323/594) (95% confident interval: [50.3- 58.3). Anaemia was moderate in 49.1% (95% confident interval: [45.1- 53.2]), and severe in 5.2% (95% confident interval: [3.7-7.3]). The prevalence of anaemia was higher among young mothers compare to adult mothers with 64.0%, 52.6% and 43.6% for mothers aged less than 20 years of age, 20-35 years of age and above 35 years of age respectively (p<0.05). By univariate and multivariate logistic regression analyses, the young age and microscopically detected malaria were independently associated with the presence of anaemia (Table 2).

**Table 1:** women basic characteristics at inclusion (n=594)

Characteristics	Items	Total	Percentage
Age (years)	< 20	136	22.9
	20-35	403	67.8
	≥ 35	55	9.3
Educational level	None	316	53.3
	Primary	86	14.5
	Secondary	191	32.2
	Plus missing*	1	-
Occupation	Unemployed	410	69.3
	Employed	182	30.7
	Self employed missing*	2	-
Parity	Primiparity	195	32.9
	Pauciparity	303	51.1
	Multiparity	95	16.2
	Missing*	1	-
Miscarriage/still birth	No	532	91.9
	Yes	47	8.1
	Missing*	15	-
BMI at inclusion (in kg/m^2^)	< 18.5	28	4.8
	≥ 18.5	559	95.2
	Missing*	7	-
HBP at inclusion	No	571	96.1
	Yes	23	3.9
Bed nets use	No	41	7.0
	Yes	545	93.0
	Missing*	8	-
Number of visits	< 4	246	41.4
	≥ 4	348	58.6
3-doses SP	No	136	22.9
	Yes	457	77.1
	Missing*	1	-
Malarial infection	No	490	82.5
	Yes	104	17.5
Temperature ≥ 37.5°C	No	519	87.4
	Yes	75	12.6

SP: sulfadoxine-pyrimethamine; CI: confidence interval; BMI: body mass index, HBP: high blood pressure; *Missing values were not included in calculations, but only presented as number

**Table 2:** prevalence and factors of anaemia in Yako health district, Burkina Faso 2019-20

Characteristics	N	Anaemia (%)	OR (95%CI)	p	aOR (95%CI)	p
**Overall**	594	54.4	-		-	
**Age categories (years)** < 20	136	64.0	1.6 [1.1 – 2.4]	0.021	1.5 [1.1 – 2.3]	0.036
20-34	403	52.6	Ref	-	Ref	-
≥ 35	55	43.6	0.7 [0.4 – 1.2]	0.21	0.7 [0.5 – 1.3]	0.31
**Educational level** None	316	57.0	1.3 [0.9 - 1.9]	0.11	-	-
Primary	86	54.6	1.2 [0.7 – 2.0]	0.45		
Secondary - plus	191	49.7	Ref			
**Occupation** Unemployed	410	53.6	0.9 [0.9 – 1.6]	0.68	-	-
Employed/self employed	182	55.5	Ref			
**Gravidity** 1	195	56.9	1.2 [0.2 – 1.6]	0.40	-	-
2 or more	398	53.3	Ref			
**Miscarriage/ stillbirth** Yes	47	46.8	0.7 [0.3 - 1.3]	0.26	-	-
No	532	55.3	Ref			
**BMI (kg/m2)** < 18.5	28	53.6	1.0 [0.9 – 2.1]	0.93	-	-
≥ 18.5	559	54.4	Ref			
**HBP at inclusion** Yes	23	54.5	O.9 [0.4 – 2.1]	0.83	-	-
No	571	52.2	Ref			
**Bed net use** No	41	56.1	1.1 [0.6 - 2.0]	0.82	-	-
Yes	545	54.3	Ref			
**SP uptake (doses)** < 3	136	58.8	1.3 [0.9 – 1.9]	0.25	-	-
≥ 3	457	53.2	Ref			
**Number of visits** < 4	246	56.9	1.2 [0.9 – 1.6]	0.86	-	-
≥ 4	348	52.6	Ref			
**Malaria** Yes	104	69.2	2.2 [1.4 - 3.4]	0.001	2.0 [1.3 - 3.2]	0.002
No	490	51.2	Ref		Ref	

SP: sulfadoxine-pyrimethamine; CI: confidence interval; HBP: high blood pressure, **Ref**: reference group, **HBP**: high blood pressure, kg/m^2^: kilogram per square meter, **OR**: Odds ratio, **aOR**: adjusted OR

## Discussion

This study examined the prevalence and factors associated of anaemia among pregnant women during their third trimester in Burkina Faso. Importantly, the study was conducted in the rural area where the transmission of malaria is highest in the country [19]. This study hypothesized that the prevalence of maternal anaemia would be reduced from 59% to 40% after two decades of the IPTp-SP policy change and daily iron supplementation among pregnant women [20]. The study results come at the right moment when the 2019 coronavirus disease (COVID-19) pandemic has substantially disrupted the health services organization in sub-Saharan Africa (SSA), and the country needs updated information on disease burden, for evidence-based interventions. In this study the reported prevalence was higher than that expected (nearly 55%), higher than the average prevalence of 35.5% in sub-Saharan Africa [16], but lower than that reported among hospitalized pregnant women in the same settings [21]. This finding confirmed the high prevalence of anaemia during pregnancy in Burkina Faso; beyond the threshold of 40% established by the WHO as severe public health problem [22]. Therefore, more efficacious interventions are needed to prevent anaemia and its consequences on pregnant women. In the literature, anaemia is usually multifactorial, with iron deficiency representing half of the causes and infections also reported as key factors in the risk of anaemia occurrence in pregnancy [23]. This study found that the presence of malaria was associated with higher prevalence of anaemia in the rural area of Burkina Faso particularly among young adolescent pregnant women. The report was similar to the finding from other settings, supporting that malaria is a key risk factor which has motivated the initiation of the WHO policy of IPTp-SP as a prevention strategy for malaria and its consequences in SSA [9,24,25]. More efforts are thus needed to prevent malaria in pregnancy in area where transmission is endemic. The study showed that nearly 25% of pregnant women did not receive the required minimum three doses of sulfadoxine pyrimethamine for the intermittent preventive treatment of malaria in pregnancy and almost 42% of women did not perform the require minimum number of four antenatal visits during their pregnancy. Thus, approaches could be developed to address these missed opportunities and the contribution of community health workers for early identification of pregnancies in the community and their referral to antenatal services could engage more pregnant women in the follow-up, and improve pregnancy safety. The study also, find that adolescent pregnant women were disproportionately affected particularly during their first pregnancies and recent studies also found that malaria prevalence was highest among this population [25-27]. Efforts could focus on reducing adolescent pregnancies through community engagement and adolescent young women sensitization, and also on the prevention of malaria infection as well as adequate detection and treatment of cases within this group [26,28]. Contrary to that was expected, the timing of antenatal visit and sulfadoxine-pyrimethamine (SP) uptake were not directly linked to an increased risk of maternal anaemia, however, late visit could reduce the number of interventions aiming to reinforce efforts to reduce malaria related anaemia in the study settings [29]. The prevalence of severe anaemia reported in the current study was higher than that reported in other sub-Saharan African setting [30,31], and this could be attributable to the spatial variations of malaria transmission among pregnant women in sub-Saharan Africa [32,33] as malaria has been identified as an important cause of severe anaemia in pregnancy, and Burkina Faso is one on the main countries severely affected in the region [34]. Adequate and early initiation of malaria prevention measures during pregnancy could reduce the risk of severe anaemia among the study population, and thus in the country at large.

**Study limitations and bias:** important study limitations are worth noting as in this study potential selection bias could have been generated because of the inclusion process which focused only on pregnant women visiting antenatal services. However, the contribution of the community health workers, belonging to the community itself, in the identification processes of pregnant women could have minimised it, by attracting some of the pregnant women who were not use to frequently visit health centres.

**Funding:** the fieldwork in this project was supported by the Clinical Research Unit of Nanoro, Burkina Faso. The funding body had no role in the design of the study and collection, analysis, and interpretation of data and in drafting the manuscript.

## Conclusion

Anaemia during pregnancy in Burkina Faso has not improved remarkably since the change of the IPTp policy and remains excessive among pregnant women particularly among adolescent pregnant women and those infected with malaria. Therefore, interventions to promote the strengthening of antenatal care and malaria preventive measures particularly among young adolescent pregnant women are needed to reduce the high level of anaemia among pregnant women in Burkina Faso.

### What is known about this topic

- *Anemia in pregnancy is a severe public health problem in Burkina Faso;*
- *Burkina Faso has implemented the World Health Organization recommended strategies to reduce the prevalence of anaemia in pregnancy*.

### What this study adds

- *Maternal anaemia prevalence remains high in the rural areas of Burkina Faso after two decades of the implementation of the intermittent preventive treatment with sulfadoxine-pyrimethamine (IPTp-SP) and daily iron supplementation strategies;*
- *Malaria remains a major risk factor for anaemia particularly among young adolescent pregnant women in the rural area of Burkina Faso;*
- *Nearly 25% of pregnant women are still not covered by the 3-doses IPTp-SP policy*.

## References

[1] World Health Organization (2011). Haemoglobin concentrations for the diagnosis of anaemia and assessment of severity.

[2] World Health Organization (2014). Comprehensive implementation plan on maternal, infant, and young child nutrition. WHO.

[3] Brabin BJ, Hakimi M, Pelletier D (2001). An analysis of anemia and pregnancy-related maternal mortality. J Nutr.

[4] Díaz-López A, Ribot B, Basora J, Arija V (2021). High and Low Haemoglobin Levels in Early Pregnancy Are Associated to a Higher Risk of Miscarriage: A Population-Based Cohort Study. Nutrients.

[5] Rahman MM, Abe SK, Rahman MS, Kanda M, Narita S, Bilano V (2016). Maternal anemia and risk of adverse birth and health outcomes in low-and middle-income countries: systematic review and meta-analysis. Am J Clin Nutr.

[6] World Health Organization Guideline Daily iron and folic acid supplementation in pregnant women.

[7] Ilboudo B, Traoré I, Méda CZ, Hien A, Kinda M, Dramaix-Wilmet M (2021). Prevalence and factors associated with anaemia in pregnant women in cascades region of burkina faso in 2012. Pan Afr Med J.

[8] Koura GK, Accrombessi MMK, Bodeau-livinec F (2012). Maternal anemia at first antenatal visit: prevalence and risk factors in a malaria-endemic area in Benin. Am J Trop Med Hyg.

[9] Sentongo P, Ba DM, Ssentongo AE, Ericson JE, Wang M, Liao D (2020). Associations of malaria, HIV, and coinfection, with anemia in pregnancy in sub-Saharan Africa?: a population-based cross-sectional study. BMC Pregnancy Childbirth.

[10] Agudelo EC, Kim HY, Musuka GN (2021). The epidemiological landscape of anemia in women of reproductive age in sub-Saharan Africa. Sci Rep.

[11] World Health Organization (2014). WHO policy brief for the implementation of intermittent preventive treatment of malaria in pregnancy using sulfadoxine-pyrimethamine (IPTp-SP).

[12] Haider BA, Olofin I, Wang M, Spiegelman D, Ezzati M, Fawzi WW (2013). Anaemia, prenatal iron use, and risk of adverse pregnancy outcomes: Systematic review and meta-analysis. BMJ.

[13] Ba DM, Ssentongo P, Kjerulff KH, Na M, Liu G, Gao X (2019). Adherence to Iron Supplementation in 22 sub-Saharan African Countries and Associated Factors among Pregnant Women: A Large Population-Based Study. Curr Dev Nutr.

[14] Karyadi E, Reddy JC, Dearden KA, Purwanti T, Mardewi, Asri E (2023). Antenatal care is associated with adherence to iron supplementation among pregnant women in selected low-middle-income-countries of Asia, Africa, and Latin America and the Caribbean regions: Insights from Demographic and Health Surveys. Matern Child Nutr.

[15] Galloway R, Dusch E, Elder L, Achadi E, Grajeda R, Hurtado E (2002). Women´s perceptions of iron deficiency and anemia prevention and control in eight developing countries. Soc Sci Med.

[16] Fite MB, Assefa N, Mengiste B (2021). Prevalence and determinants of Anemia among pregnant women in sub-Saharan Africa: a systematic review and Meta-analysis. Arch Public Health.

[17] Ministère de la Santé Burkina Faso (2018). Annuaire statistique de sante 2017. Ministère de la santé Burkina Faso.

[18] Ministère de la Santé Burkina Faso/DGESS. Annuaire statistique 2018.

[19] Ouédraogo A, Tiono AB, Diarra A, Sanon S, Yaro JB, Ouedraogo E (2013). Malaria Morbidity in High and Seasonal Malaria Transmission Area of Burkina Faso. PLoS One.

[20] He Z, Bishwajit G, Yaya S, Cheng Z, Zou D, Zhou Y (2018). Prevalence of low birth weight and its association with maternal body weight status in selected countries in Africa: a cross-sectional study. BMJ Open.

[21] Ouedraogo I, Zamané H, Sawadogo AY, Kiemtoré S, Kain DP, Ouattara A (2019). Anaemia in Pregnancy in an African Setting after Preventive Measures. Open J Obstet Gynecol.

[22] World Health Organization Anaemia in women and children. WHO.

[23] Valero De Bernabé J, Soriano T, Albaladejo R, Juarranz M, Calle ME, Martínez D (2004). Risk factors for low birth weight: A review. Eur J Obstet Gynecol Reprod Biol.

[24] Adam I, Ibrahim Y, Elhardello O (2018). Prevalence, types and determinants of anemia among pregnant women in Sudan: A systematic review and meta-analysis. BMC Hematol.

[25] Mlugu EM, Minzi O, Kamuhabwa AAR, Aklillu E (2020). Prevalence and correlates of asymptomatic malaria and anemia on first antenatal care visit among pregnant women in Southeast, Tanzania. Int J Environ Res Public Health.

[26] Pons-Duran C, Mombo-Ngoma G, Macete E, Desai M, Kakolwa MA, Zoleko-Manego R (2022). Burden of malaria in pregnancy among adolescent girls compared to adult women in 5 sub-Saharan African countries: A secondary individual participant data meta-analysis of 2 clinical trials. PLoS Med.

[27] Osarfo J, Ampofo GD, Tagbor H (2022). Trends of malaria infection in pregnancy in Ghana over the past two decades: a review. Malar J.

[28] Bhalla D, Cleenewerck L, Kalu SO, Gulma KA (2019). Malaria Prevention Measures among Pregnant Women?: a population-based survey in Nnewi, Nigeria. Sci World J.

[29] Saapiire F, Dogoli R, Mahama S (2022). Adequacy of antenatal care services utilisation and its effect on anaemia in pregnancy. J Nutr Sci.

[30] Abdallah F, John SE, Hancy A, Paulo HA, Sanga A, Noor R (2022). Prevalence and factors associated with anaemia among pregnant women attending reproductive and child health clinics in Mbeya region, Tanzania. PLOS Glob Public Health.

[31] Sumaila I, Manu A, Hallidu M (2022). Prevalence and associated factors of anaemia among antenatal care attendants in the Kintampo municipality. Pan African Med J One Heal.

[32] Gore-Langton GR, Cano J, Simpson H, Tatem A, Tejedor-Garavito N, Wigley A (2022). Global estimates of pregnancies at risk of Plasmodium falciparum and Plasmodium vivax infection in 2020 and changes in risk patterns since 2000. PLOS Glob Public Health.

[33] van Eijk AM, Hill J, Noor AM, Snow RW, ter Kuile FO (2015). Prevalence of malaria infection in pregnant women compared with children for tracking malaria transmission in sub-Saharan Africa: A systematic review and meta-analysis. Lancet Glob Health.

[34] Shulman CE, Dorman EK, Bulmer JN (2002). Malaria as a cause of severe anaemia in pregnancy. Lancet.

